# Caffeine and Its Interactions with Antiseizure Medications—Is There a Correlation between Preclinical and Clinical Data?

**DOI:** 10.3390/ijms242417569

**Published:** 2023-12-17

**Authors:** Barbara Miziak, Barbara Błaszczyk, Magdalena Chrościńska-Krawczyk, Stanisław J. Czuczwar

**Affiliations:** 1 Department of Pathophysiology, Medical University of Lublin, 20-090 Lublin, Poland; barbara.miziak@umlub.pl; 2 Faculty of Medical Sciences, Lipinski University, 25-734 Kielce, Poland; barbarablaszczyk@op.pl; 3 Department of Child Neurology, Medical University of Lublin, 20-093 Lublin, Poland; madziachr@wp.pl

**Keywords:** caffeine, methylxanthines, antiseizure medications, epilepsy, seizures

## Abstract

Experimental studies reveal that caffeine (trimethylxanthine) at subconvulsive doses, distinctly reduced the anticonvulsant activity of numerous antiseizure medications (ASMs) in rodents, oxcarbazepine, tiagabine and lamotrigine being the exceptions. Clinical data based on low numbers of patients support the experimental results by showing that caffeine (ingested in high quantities) may sharply increase seizure frequency, considerably reducing the quality of patients’ lives. In contrast, this obviously negative activity of caffeine was not found in clinical studies involving much higher numbers of patients. ASMs vulnerable to caffeine in experimental models of seizures encompass carbamazepine, phenobarbital, phenytoin, valproate, gabapentin, levetiracetam, pregabalin and topiramate. An inhibition of R-calcium channels by lamotrigine and oxcarbazepine may account for their resistance to the trimethylxanthine. This assumption, however, is complicated by the fact that topiramate also seems to be a blocker of R-calcium channels. A question arises why large clinical studies failed to confirm the results of experimental and case-report studies. A possibility exists that the proportion of patients taking ASMs resistant to caffeine may be significant and such patients may be sufficiently protected against the negative activity of caffeine.

## 1. Introduction

Caffeine, structurally a 1,3,7-trimethylxanthine, is a nonselective antagonist of adenosine receptors [1]. All types of adenosine receptors (A1, A2A, A2B, A3) are coupled to G proteins [2] and their activation differentially affects the activity of adenylyl cyclase and thus, the neuronal concentration of cAMP. Whilst stimulation of A1 receptors by adenosine results in the reduced activity of adenylyl cyclase [3], an opposite effect is evident with A2 receptors [4,5]. A1 receptors are widely distributed throughout the central nervous system (brain stem, cerebellum, cortex, hippocampus, spinal cord) and are also present in peripheral tissues [2,6]. It is widely documented that the stimulation of A1 receptors is associated with anticonvulsant activity among other centrally mediated actions (anxiolytic effects, inhibition of locomotor activity, sedation) [2]. A2 adenosine receptors are not so widely distributed compared to A1 receptors and are mainly found in the striatum and other brain structures with high dopamine concentrations [7]. The data on A2 receptors and seizure activity are unequivocal, reporting both anti- and proconvulsant effects [8,9]. Interestingly, the activation of A3 receptors resulted either in anticonvulsant effects or lack of seizure threshold modulation, depending on the seizure model used [8,10].

Caffeine is frequently ingested as a stimulant in the form of beverages (tea, coffee) or soft drinks. Moreover, it may be contained in a variety of medicines (appetite stimulants, analgesics or some antivirals). A question arises whether the consumption of beverages containing caffeine may provide pharmacologically relevant concentrations. For instance, teenagers, according to Bernstein et al. [11] may ingest circa 200 mg of caffeine daily, on average. Importantly, no significant difference was observed in the amount of ingested trimethylxanthine in both caffeine dependent and non-dependent subjects [11]. The maximum daily consumption of caffeine should not exceed 400 mg as recommended by the European Food Safety Authority [12]. The content of caffeine per cup is strictly related to the preparation methods. An American coffee cup (filtration) contains 316 mg of caffeine, Turkish (boiling)—112 mg, whilst Espresso (extraction under pressure)—64 mg [12]. Heavy coffee drinkers may consume around five cups daily while very heavy ones—even more than six [13]. All these data clearly indicate that, especially in heavy coffee drinkers, caffeine is likely to reach pharmacologically active concentrations.

Caffeine in a high-dose range, usually exceeding 400 mg/kg in mice [14] or 150 mg/kg in rats [15], produced potent seizure activity. Trimethylxanthine, in much lower doses, was also documented to lower the convulsive threshold in some seizure models. For instance, the seizure threshold was decreased in rats following acute or chronic administration of caffeine (at 50 or 80 mg/kg) in pentylenetetrazol-induced seizures [16]. Also, a reduction of the threshold by caffeine at a dose of 92.4 mg/kg was reported in mice [14]. However, according to Bankstahl et al. [17] threshold alteration was not observed in rats after high doses of caffeine (60–80 mg/kg) in the intravenous pentylenetetrazol test. Noteworthy, several of the caffeine-treated rats developed status epilepticus despite termination of pentylenetetrazol infusion at the first clonic seizure, which was not observed in controls [17]. Caffeine, on the other hand, lowered the threshold for maximal electroconvulsions in rats. This effect, however, was not confirmed for caffeine (46.2 mg/kg) in mice [14]. Nonetheless, caffeine or theophylline (dimethylxanthine; both at 100 mg/kg orally in rats) distinctly potentiated the EEG and behavioral effects of pentylenetetrazol given at the subconvulsive dose of 30 mg/kg [18].

Most patients with epilepsy receive antiseizure medications (ASMs) and obviously some of them may be coffee consumers [19]. A possibility arises that caffeine-induced adenosine receptor blockade may affect the protection offered by ASMs. This review includes studies related to this problem both dealing with the experimental and clinical aspects of possible caffeine influence on the protective activity of ASMs.

## 2. Search Strategy and Selection Criteria

The article search was conducted in PubMed, Google Scholar and Web of Science databases and the keywords were: antiepileptic drugs (or ASMs), caffeine and animal seizure tests for experimental data as well as antiepileptic drugs (ASMs), coffee (or caffeine or energetic drinks) and seizure frequency for clinical data. The search was preferentially focused on articles published in the time frame of 1985–2023.

## 3. Caffeine and the Anticonvulsant Activity of ASMs

### 3.1. Data from Electroconvulsive Tests in Rodents—Maximal Electroshock Test (MES) or Maximal Electroshock Threshold Test (MEST)

MES or MEST tests are reliable models of human generalized tonic-clonic seizures [20]. Usually, available data were extracted from MES tests in mice unless stated otherwise.

In a relatively high dose of 200 mg/kg, acute caffeine impaired the protective activity of conventional ASMs (diazepam, phenobarbital, phenytoin) in rats, valproate being an exception to the rule. A possibility of pharmacokinetic interactions was not considered [21].

The trimethylxanthine was also studied in lower dose ranges (starting from 92.4 mg/kg). The most susceptible ASMs was phenobarbital whose anticonvulsant activity was lowered by caffeine even at 11.55 mg/kg. The ED50 of phenobarbital against MES of 19.5 mg/kg was almost doubled to 38 mg/kg by caffeine at 92.4 mg/kg. The protection offered by phenytoin against MES with an ED50 of 12 mg/kg was also considerably reduced by caffeine at 46.2 and 92.4 mg/kg, which was reflected by increases in its ED50 values to 17 and 24 mg/kg, respectively. When administered at 46.2 and 92.4 mg/kg, caffeine distinctly reduced the anticonvulsant activity of carbamazepine and valproate, elevating their ED50s against MES at the highest dose from 13 to 20.5 mg/kg and from 270 to 420 mg/kg, respectively. A pharmacokinetic verification revealed that almost all interactions were free from pharmacokinetic events—only the plasma concentration of phenytoin was reduced in the presence of caffeine at 92.4 mg/kg [14].

The protective activity of ASMs was also studied in the chronic administration of caffeine to find out whether this obviously negative activity of acute caffeine is subject to tolerance. When the trimethylxanthine was injected in a dose of 11.55 mg/kg twice daily for 14 days, the ED50 of carbamazepine was significantly increased, the effect being not observed following the acute or 3-day administration of caffeine in this dose. When given at 23.1 mg/kg (acutely, for 3 days as a subchronic treatment or 14 days as a chronic administration), caffeine reduced the anticonvulsant activity of phenytoin, carbamazepine being affected by the chronic trimethylxanthine only. In no case did either acute or chronic trimethylxanthine affect the plasma concentrations of these ASMs [14]. Similar effects were evident when subchronic or chronic caffeine (at 23.1 mg/kg) was tested in combination with phenobarbital or valproate whose protective activity was reduced, and no pharmacokinetic interactions were observed [14]. Interestingly, after 24 or 72 h withdrawal of caffeine, the protective action of all of the above tested ASMs returned to the control values but a challenge dose of caffeine (23.1 mg/kg) led to a strong impairment of the anticonvulsant effects of these ASMs, in some cases being more pronounced when compared to its acute or chronic administration [14].

Considering newer ASMs, acute and chronic caffeine (23.1 and 46.2 mg/kg) impaired the anticonvulsant effect of topiramate and in the same doses reduced the gabapentin-produced elevations of the electroconvulsive threshold in the MEST. The free plasma concentrations of either topiramate or gabapentin were not affected by caffeine. Both, acute and chronic caffeine (23.1 and 46.2 mg/kg) potentiated gabapentin-induced impairment of motor coordination but an opposite effect was noted for topiramate neurotoxicity—chronic caffeine at 46.2 mg/kg reduced the motor impairment produced by this ASM [22]. As for pregabalin, this ASM was also susceptible to caffeine (23.1 mg/kg)—its protective ED50 was significantly reduced and moreover, caffeine prevented pregabalin-induced motor impairment and did not affect pregabalin’s total brain concentration [23]. Caffeine (46.2 and 69.3 mg/kg) also reduced the protective activity of levetiracetam (500 mg/kg) against MEST but the combined treatment did not result in any disturbance of motor performance. Levetiracetam was without influence on the total brain concentration of caffeine and the trimethylxanthine did not affect the total brain concentration of levetiracetam [24]. Another newer ASM, felbamate, was partially resistant to the negative influence of caffeine, because the trimethylxanthine was capable of reducing its anticonvulsant effect at a high dose of 161.7 mg/kg [14]. Strikingly, acute or chronic caffeine at 46.2 mg/kg proved completely inactive on the anticonvulsant effects of oxcarbazepine and lamotrigine and in the MEST, it did not affect the tiagabine-induced increase in the convulsive threshold. Although the total brain concentrations of oxcarbazepine or lamotrigine were not influenced by caffeine, the chronic trimethylxanthine administration elevated the total brain level of tiagabine. In no case did caffeine affect the neurotoxic potential of oxcarbazepine, lamotrigine or tiagabine in terms of motor coordination and long-term memory [14].

### 3.2. Data from Pentylenetetrazol-Induced Clonic Seizure Activity

Pentylenetetrazol-induced clonic seizures in rodents may be regarded as a model of human myoclonic seizures [25]. Initial studies on the interactions between ASMs and caffeine were conducted without pharmacokinetic verifications [21,26]. Goto et al. [26] revealed that the anticonvulsant activity of diazepam (0.5 mg/kg) against pentylenetetrazol in mice was attenuated by caffeine (200 mg/kg). Using the same high dose of caffeine, Kulkarni et al. [21] showed that trimethylxanthine distinctly reduced the protective activity of diazepam and phenobarbital but not that of ethosuximide or valproate. However, caffeine in the dose range of 69.3–92.4 mg/kg diminished the anticonvulsant action of ethosuximide in mice which was reflected by the increase in its respective ED50 values from 127.7 to 182.3 and 198.3, respectively. Noteworthy, trimethylxanthine at 92.4 mg/kg decreased the convulsive threshold for pentylenetetrazol—its CD50 (a convulsive dose required to induce clonic seizures in 50% of mice) was reduced from 69.5 to 51.7 mg/kg [14]. However, at this dose caffeine was without effect upon the anticonvulsant activity of clonazepam, phenobarbital and valproate and moreover, it did not affect the total brain concentrations of all ASMs studied. Either alone or in combination with ASMs, caffeine (92.4 mg/kg) did not influence the motor coordination of mice [14]. The effects of caffeine on the convulsive threshold and protective efficacy of ASMs are shown in Table 1 and Table 2.

## 4. Do Other Methylxanthines Share Caffeine’s Propensity to Affect the Anticonvulsant Activity of ASMs?

Theophylline (1,3-dimethylxanthine) has been used as a medication for the management of acute and chronic asthma [27]. Applied in the form of aminophylline (theophylline2. ethylenediamine), it induced severe seizure activity in mice at the dose of 280 mg/kg. Among a variety of ASMs, only phenobarbital and diazepam were able to fully protect the animals against clonic seizure activity [28]. Similarly to caffeine, the dimethylxanthine (at subconvulsive doses of 50 mg/kg or lower) distinctly attenuated the anticonvulsant activity of many conventional ASMs (carbamazepine, diazepam, phenobarbital, phenytoin, valproate), both after acute and chronic administration [14]. Interestingly, another methylxanthine derivative, pentoxifylline induced clonic seizure activity in mice with a CD50 of 250 mg/kg whilst diprophylline up to 1000 mg/kg was inactive in this regard. Regarding their influence on conventional ASMs, pentoxifylline (at 66.3–132.5 mg/kg) and diprophylline (at 242.1 mg/kg) were capable of reducing the anticonvulsant activity of phenytoin against MES in mice. However, the protective effects of the remaining ASMs (carbamazepine, phenobarbital, valproate) were not affected by these methylxanthines [14].

## 5. Do the Experimental Results Translate into Clinical Studies?

### 5.1. Studies Pointing to a Relationship between Caffeine Intake and Seizure Activity

Only clinical studies performed on low numbers of patients, mainly case-reports, seem to confirm the experimental findings on the negative role of caffeine in the anticonvulsant efficacy of ASMs. A male patient, forty nine years of age, with a history of epilepsy for 36 years (absence, atonic, and myoclonic seizures) experienced a potent increase in atonic and myoclonic seizure frequency despite ASMs’ therapeutic blood levels, with no changes in stress level or sleep patterns [29]. Also, no concurrent diseases or additional medicines were evident. A replacement of conventional ASMs to newer ones was even considered. However, analyzing the patient’s diet revealed that he, trying to avoid caloric beverages, had started to drink more than 1.5 L (4 pints) of diet iced tea daily. After 2 months, the patient substituted the diet iced tea for a decaffeinated beverage and his seizure frequency returned to baseline [29]. Another case report presents a male patient (forty years old) who started seizure activity at the age of eleven in the form of simple and complex focal seizures [30]. A couple of ASMs were tried but the patient was never seizure-free. At the time of the study, the patient, prescribed carbamazepine, used to drink up to 2 L (5 pints) of coffee and reported circa five simple focal seizures daily and one complex partial seizure per week. After quitting coffee drinking, his seizure frequency was sharply reduced to one simple focal seizure daily, complex partial seizures being no longer present [30]. Mackow et al. [31] presented a female patient (33 years old) with a history of temporal lobe epilepsy since infancy. After having tried a variety of ASMs, she became compliant to carbamazepine, levetiracetam and zonisamide. Following an unsuccessful surgery, she underwent the implantation of a responsive neurostimulation system recognizing the onset of seizures. Nonetheless, the patient suffered from recurrent episodes of status epilepticus and interestingly, epileptiform discharges would start to emerge from Fridays until Sundays. It turned out that during the weekends, the patient used to consume more than 1.5 L of caffeinated soda. When she stopped her habit on weekends, a sharp decrease in the mean number of epileptiform discharges was evident—from 841 to 107 daily on Fridays, Saturdays and Sundays which was associated with a reduction in seizure activity and no recurrence of status epilepticus [31]. More patients with epilepsy were analyzed who were questioned about their habit of drinking coffee—out of a hundred patients, 78 responded positively [32]. As a matter of fact, 71 patients did not find any relationship between their habit and seizure frequency. Noteworthy, 49 of them would drink up to two cups and 22 patients—more than two cups of coffee daily. However, in seven patients ingesting 4–5 cups of coffee, a considerable increase in seizure activity was evident. Quitting the habit in some patients, restored their seizure frequency to baseline [32].

### 5.2. Studies Showing Mainly No Association between Dietary Caffeine and Seizure Precipitation

The role of caffeine as a factor precipitating seizures was investigated by Samonsen et al. [33]. The authors characterized the caffeine consumption in standard units—one unit was equal to one cup of coffee, two cups of tea or three caffeinated soft drinks. The patients (a total of 174 patients, 57% males and mean age of 42.6 years) were divided into four groups: 1. no caffeine, 2. low intake (≤3 units), 3. moderate consumption (˂3–≥6 units), and 4. high consumption (˃6 units). The major epilepsy types were: focal seizure onset (89 patients), genetic generalized epilepsies (22), and absences (5). Overall, about 67% of the patients were diagnosed with epilepsy. The results indicate that there was no difference between the caffeine consumption 24 h before the seizure and its intake on a day without seizures [33]. Dvoretzky et al. [34] examined a number of health and lifestyle parameters in 116,608 nurses, including epilepsy (or seizures) and caffeine consumption. About 50% of participants would consume more than 200 mg of caffeine daily, with a mean of 437 mg. There was no association with the risk of seizure or epilepsy (N = 246) and caffeine intake which was comparable for the total cohort [34]. A detailed analysis of the influence of coffee consumption on seizure frequency in 619 patients with drug-resistant focal epilepsy was carried out by Bourgeois-Vionnet [35]. Coffee intake was classified into four groups: no consumption, rare (˂1 cup weekly–3 cups weekly), moderate (4 cups weekly–3 cups daily), and high (˃4 cups daily). Evidently, caffeine intake was without effect upon the seizure frequency as regards all seizure types. Strikingly, patients with moderate caffeine intake had a lower number of focal bilateral clonic-tonic seizures when compared to other groups. However, in those with high caffeine consumption (more than 4 cups of coffee daily), the trimethylxanthine favored the occurrence of focal bilateral clonic-tonic seizures [35]. The influence of caffeine on seizure frequency is depicted in Table 3.

## 6. Discussion 

Adenosine A1-receptor-mediated events are clearly involved in anticonvulsant activity in many models of seizures, including drug-resistant seizures [36]. Thus, the question may be posed whether the negative impact of caffeine on the efficacy of ASMs may be dependent on the A1 receptor blockade. This possibility seems unlikely, at least for electroconvulsions, as there are data indicating that the non-xanthine A1 receptor antagonist, 5-amino-9-chloro-2-(2-furyl)-1,2,4-triazolo[1,5-c]quinazoline (CGS 15943A), failed to affect the protective activity of conventional ASMs in the MES test (carbamazepine, phenobarbital and valproate) which were vulnerable to caffeine [14]. After all, there are data indicating that carbamazepine actually behaved as an antagonist at adenosine A1 receptors in hippocampal CA1 pyramidal cells and yet, this ASM was vulnerable to caffeine [37]. The only exception to the rule was phenytoin whose anticonvulsant effect was reduced by CGS 15943A and regarding this ASM, the blockade of adenosine A1 receptors by caffeine, apart from non-receptor mechanisms, may participate in the negative impact of the trimethylxanthine. As concerns some newer ASMs, there is no evidence whether caffeine may reduce their protective efficacy against seizure activity via the A1 receptor-related mechanism.

Apart from the adenosine receptor antagonism, trimethylxanthine may also act via other mechanisms. Methylxanthines, although at high doses, were documented to inhibit phosphodiesterases [38]. This mechanism, however, also seems unlikely in the negative impact of caffeine on a variety of ASMs as other already mentioned methylxanthines, pentoxifylline and diprophylline, were only able to reduce the anticonvulsant activity of phenytoin, exerting no effect of the protection offered by other conventional ASMs [14]. Nevertheless, considering the effects of the non-xanthine A1 receptor antagonist [37] and those of pentoxifylline or diprophylline on the anticonvulsant effect phenytoin, the A1 receptor blockade is very likely to participate in the reduction of phenytoin’s antiseizure activity. A possibility arises that the negative interaction of caffeine with a number of ASMs may involve the ryanodine receptor-mediated release of Ca2+ from the endoplasmic reticulum [39,40]. This mechanism, however, may be rejected as carbamazepine and valproate inhibited epileptiform activity in hippocampal slices produced by caffeine as a consequence of ryanodine receptor activation [40].

Next, a question regarding the effects of caffeine upon the protective effects of ASMs may be posed. Why is trimethylxanthine unable to reduce the anticonvulsant action of all tested ASMs? Probably because some mechanisms of action of the caffeine resistant ASMs may be not shared by the vulnerable ones. The ASMs whose anticonvulsant efficacy in the MES test in mice was reduced by caffeine are: carbamazepine, phenobarbital, phenytoin, valproate, gabapentin, levetiracetam, pregabalin and topiramate. Their anticonvulsant activity was diminished by caffeine at its high dose. This clearly indicates that the voltage-operated sodium channel blockade, enhancement of GABA-mediated events, binding to α2δ or SV2A sites and blockade of AMPA or NMDA receptors [41] do not determine the resistance to the negative effect of caffeine. The ASMs resistant to caffeine (lamotrigine and oxcarbazepine) possess a mechanism of action which is evidently not shared by the ASMs susceptible to caffeine. Lamotrigine and oxcarbazepine (and its active metabolite: GP 47779), apart from the mechanisms shared by other ASMs challenged with caffeine, are blockers of R-calcium channels [42,43,44], which may account for their resistance to caffeine. R-calcium channels are widely distributed in the central nervous system, including the cortex, thalamus and cerebellum and are associated with neurotransmitter release [42]. It is very probable that oxcarbazepine, by blocking these channels, leads to a considerable inhibition of glutamate release [45,46]. The interaction of lamotrigine with R-calcium channels seems of pivotal importance for the expression of its anticonvulsive activity, at least against kainate-induced convulsions in mice [44]. While lamotrigine protected against this seizure type in naïve animals, this ASM even potentiated kainate-induced seizure activity and increased neurodegeneration in hippocampal CA1 neurons in R-type channel deficient mice. Interestingly, lacosamide, showing no interaction with R-type calcium channels, exerted a protective activity in both groups of mice [44]. However, one ASM out of those vulnerable to the negative effect of caffeine, topiramate, has been also shown to interact with R-calcium channels. At a relevant concentration of 50 microM, this ASM inhibited the cholinergic-dependent plateau potentials in CA1 pyramidal neurons, as shown by current-clamp mode [47]. Nevertheless, topiramate did not affect calcium spikes under control conditions [47] and this fact could probably account for its vulnerability to caffeine. Tiagabine, as an ASM inhibiting the GABA GAT1 transporter and thus elevating the synaptic concentration of GABA [41], was resistant to caffeine. This may indicate that not all GABA enhancers (valproate or topiramate [41]) share vulnerability to trimethylxanthine.

Evidently, experimental evidence indicates that caffeine failed to diminish the anticonvulsant activity of valproate against pentylenetetrazol-induced convulsions [14,21] while for other ASMs (benzodiazepines, phenobarbital, ethosuximide) the data were ambiguous [14,21]. The differential effect of caffeine on the anticonvulsant action of valproate depending upon the model of seizures speaks for participation of different mechanisms determining the susceptibility of ASMs to trimethylxanthine.

In their broad and comprehensive review on caffeine, ASMs and seizure activity, van Koert et al. [48] also point to some beneficial effects of trimethylxanthine at low doses (5–20 mg/kg) in this respect as for instance, protection against chemically induced convulsions or electroconvulsions. There are also results available that point to the protective activity of caffeine against SUDEP in an animal model as well as against ASM-induced neurotoxicity in developing rats [48]. As regards the negative effects of caffeine on the anticonvulsant effects of a number of ASMs in animal models of seizures, they point to a very important issue of the different metabolism of trimethylxanthine in rodents and humans, which must be taken into consideration when translating the experimental data to clinical conditions. Caffeine metabolism in humans may be variable as there are polymorphisms of the CYP1A2 isoform of cytochrome P450 and N-acetyltransferase 2 [49]. The main human metabolites of caffeine are paraxanthine, theobromine and theophylline whilst in mice, theobromine and theophylline are found in trace quantities [49,50]. It is also of importance that caffeine doses in animal research are higher than those resulting from caffeine consumption [48], however, they are still significantly below the convulsive doses of trimethylxanthine [14].

**Figure 1:**
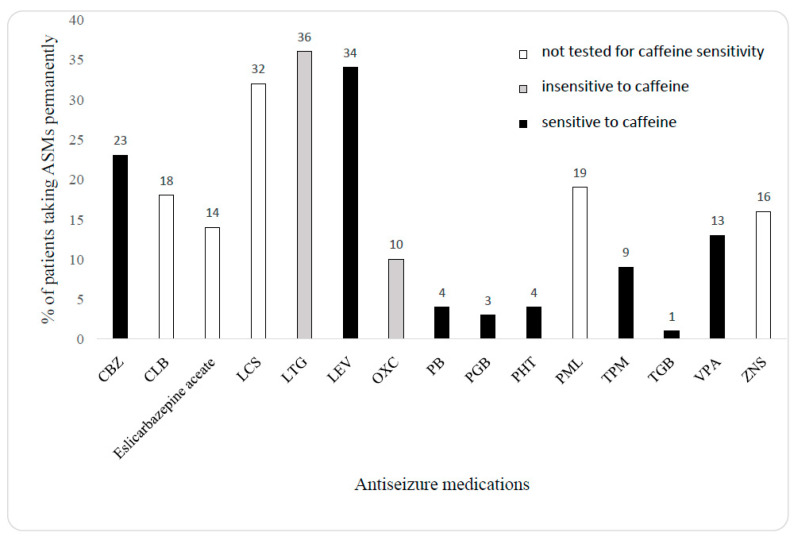
Proportions of patients taking ASMs (based on data from ref. [42]). CBZ—carbamazepine, CLB—clobazam, LCS—lacosamide, LTG—lamotrigine, LEV—levetiracetam, OXC—oxcarbazepine, PB—phenobarbital, PGB—pregabalin, PHT—phenytoin, PML—perampanel, TPM—topiramate, TGB—tiagabine, ZNS—zonisamide.

Clinical data, mainly based on small or case-report studies, support the results of experimental studies. A very high ingestion of caffeine-containing drinks was evident and generally, a caffeine-induced increase in seizure frequency would return to baseline following a change in the drinking habit. Quite different results were generally obtained from studies incorporating more than a hundred patients. Even the moderate consumption of coffee had a beneficial effect on the occurrence of focal bilateral clonic-tonic seizures. However, in the same study, the high intake of caffeine favored this seizure type [35]. Nonetheless, considering all types of seizures, these authors did not find any deleterious influence of trimethylxanthine on seizure frequency [35]. A partial explanation of this effect may be based upon detailed ongoing antiseizure medications, showing that 46% of patients received lamotrigine (36%) or oxcarbazepine (10%; Figure 1). This indicates that almost half of patients in this study (46%) could be protected against caffeine, based on the results of experimental studies. However, the data, on how the drugs were allocated in different caffeine consumption groups, are not available. Considering that some patients received more than one ASM, the proportion of patients taking caffeine-resistant ASMs could be significant. An important conclusion may be drawn from case-report studies—it seems that the consumption of high quantities of caffeine-containing drinks or a sudden increase in the ingestion of caffeine may in fact be responsible for the significant increase in seizure frequency. After all, such isolated changes in studies conducted on hundreds of patients may not fully affect the final results.

## 7. Final Conclusions

Caffeine at usually high but subconvulsive doses distinctly reduced the anticonvulsive potential of many ASMs in animal models of seizures. It is very likely that caffeine-induced A1 adenosine receptor blockade does not seem to participate in this negative effect. Only in the case of phenytoin, the A1 receptor blockade might be involved.

Case-reports support the results of experimental studies whilst large clinical studies usually find no correlation between caffeine intake and seizure frequency. Nonetheless, patients with epilepsy should avoid caffeine intake in high quantities.

## Figures and Tables

**Table 1:** Caffeine and the protective efficacy of ASMs.

ASM	Initial Doses of ASMs (mg/kg)	Caffeine			Seizure Model	Animal Model	ASM Efficacy	Bibliography
		Dose of Caffeine (mg/kg)	Acute	Chronic				
Carbamazepine	13	46.2	+	NT	MES	mouse	↓	[14]
	13	92.4	+	NT	MES	mouse	↓	[14]
	15	11.55	NT	+	MES	mouse	↓	[14]
	15	11.55	+	NT	MES	mouse	0	[14]
	15	23.1	+	NT	MES	mouse	↓	[14]
	15	23.1	NT	+	MES	mouse	↓	[14]
Diazepam	0.5	200	+	NT	PTZ	mouse	↓	[26]
	1	200	+	NT	PTZ	rat	↓	[21]
	10	200	+	NT	MES	rat	↓	[21]
Ethosuximide	127.7	69.3	+	NT	PTZ	mouse	↓	[14]
	127.7	92.4	+	NT	PTZ	mouse	↓	[14]
	200	200	+	NT	PTZ	mouse	0	[21]
Felbamate	110	161.7	+	NT	MES	mouse	↓	[14]
Clonazepam	0.026	92.4	+	NT	PTZ	mouse	0	[14]
Lamotrigine	7.5	46.2	+	+	MES	mouse	0	[14]
	7.5	23.1	+	+	MES	mouse	0	[14]
Oxcarbazepine	13	23.1	+	+	MES	mouse	0	[14]
	13	46.2	+	+	MES	mouse	0	[14]
Phenobarbital	10	200	+	NT	MES	rat	↓	[21]
	19.5	92.4	+	NT	MES	mouse	↓	[14]
	17.8	23.1	+	+	MES	mouse	↓	[14]
	11.4	92.4	+	NT	PTZ	mouse	0	[14]
	10	200	+	NT	PTZ	rat	↓	[14]
Phenytoin	12	92.4	+	NT	MES	mouse	↓	[14]
	12	46.2	+	NT	MES	mouse	↓	[14]
	20	200	+	NT	MES	rat	↓	[21]
Pregabalin	379.3	23.1	+	NT	MES	mouse	↓	[23]
Topiramate	44.8	23.1	+	+	MES	mouse	↓	[22]
		46.2	+	+	MES	mouse	↓	[22]
Valproate	300	200	+	NT	MES	rat	0	[21]
	270	46.2	+	NT	MES	mouse	↓	[14]
	273	23.1	NT	+	MES	mouse	↓	[14]
	270	92.4	+	NT	MES	mouse	↓	[14]
	130.7	92.4	+	NT	PTZ	mouse	0	[14]
	300	200	+	NT	PTZ	mouse	0	[21]

↓—ASM efficacy decreased; 0—no effect upon ASM efficacy; ASM—antiseizure medication; +—tested; NT—not tested; MES—maximal electroshock; PTZ—pentylenetetrazol.

**Table 2:** Influence of caffeine upon the convulsive threshold and ASM-induced elevations of the convulsive threshold.

ASM	Dosage of ASMs (mg/kg)	Caffeine			Seizure Model	Animal Model	Convuslive Threshold	Bibliography
		Dose of Caffeine (mg/kg)	Acute	Chronic				
NT	NT	50	+	NT	PTZ	rat	↓	[16]
		60	+	NT	PTZ	rat	0	[17]
		92.4	+	NT	PTZ	mouse	↓	[14]
		92.4	+	NT	PTZ	rat	↓	[14]
		80	+	NT	MEST	rat	↓	[17]
		80	NT	+	PTZ	rat	↓	[16]
Gabapentin	200	23.1	NT	+	MEST	mouse	↓	[22]
	200	46.2	+	+	MEST	mouse	↓	[22]
Levetiracetam	500	46.2	+	NT	MEST	mouse	↓	[24]
	500	69.3	+	NT	MEST	mouse	↓	[24]
Tiagabine	4.9	23.1	+	+	MEST	mouse	0	[14]
	4.9	46.2	+	+	MEST	mouse	0	[14]

↓—decreased; 0—no effect; ASM—antiseizure medication; MEST—maximal electroshock threshold test; PTZ—pentylenetetrazol; +—tested; NT—not tested.

**Table 3:** Ingestion of caffeine and seizure activity in patients with epilepsy.

Study		Age of Patients (Years)	Gender	Type of Seizures	Exposure to Increased Doses of Caffeine	Seizure Frequency	Seizure Frequency after Withdrawal of Caffeine Source	Bibliography
Case Reports	Clinical Study							
+		49	M	absence, atonic, and myoclonic seizures	+	↑	↓	[29]
+		40	M	simple and complex focal seizures	+	↑	↓	[30]
+		33	F	temporal lobe epilepsy recurrent episodes of status epilepticus	+	↑	↓	[31]
	+	42.6 (mean of years)	M—57% F—43%	focal seizure onset (89 patients) and genetic generalized epilepsies (22) and absences (5)	+	0	0	[33]
	+	25–42	F	self-reported seizure or epilepsy	+	0	0	[34]
	+	18–77	M, F	drug-resistant focal epilepsy, focal bilateral clonic-tonic seizures	moderate caffeine	↓	No data	[35]
			M, F	drug-resistant focal epilepsy, focal bilateral clonic-tonic seizures	high caffeine consumption	↑	↓	[35]

+—present; ↑—increase in convulsive activity; ↓—decrease in convulsive activity; 0—no effect; M—male; F—female.
